# Cardiovascular hemodynamic response to peak exercise in individuals with multiple sclerosis

**DOI:** 10.14814/phy2.70150

**Published:** 2024-12-26

**Authors:** Brooks A. Hibner, Natalia S. Lima, Sara R. Sherman, Robert W. Motl, Julio A. Chirinos, Shane Phillips, Philip S. Clifford, Anthony T. Reder, Tracy Baynard, Bo Fernhall

**Affiliations:** ^1^ Integrative Physiology Laboratory University of Illinois at Chicago Chicago Illinois USA; ^2^ Hospital of the University of Pennsylvania Perelman School of Medicine at the University of Pennsylvania Philadelphia Pennsylvania USA; ^3^ Department of Physical Therapy University of Illinois at Chicago Chicago Illinois USA; ^4^ Department of Neurology University of Chicago Medicine Chicago Illinois USA; ^5^ Department of Exercise and Health Sciences University of Massachusetts Boston Boston Massachusetts USA

**Keywords:** aerobic capacity, hemodynamics, neurological disorders

## Abstract

Multiple sclerosis (MS) is a chronic neurological condition resulting in decreased aerobic capacity (peak VO_2_). The hemodynamic responses to peak exercise in MS are unknown. Further, it is unknown if the hemodynamic responses are due to disease or fitness. Therefore, the purpose was to compare hemodynamic response to peak exercise between individuals with and without MS, with similar peak VO_2_. Individuals with MS (*n* = 21) and CON (*n* = 21) underwent maximal incremental cycle exercise test to assess peak aerobic capacity (peak VO_2_). Heart rate, stroke volume, cardiac output, and blood pressure were obtained every other minute of the exercise test. There were no significant group differences in peak VO_2_. All hemodynamic variables increased similarly from baseline to peak exercise in both MS and CON. There was a significant group by time interaction for HR in individuals with MS (*p* < 0.01), accounted for by age, but no group by time interactions in MAP (*p* = 0.78), SV (*p* = 0.11), or Q (*p* = 0.86). Our findings suggest that individuals with and without MS, with similar peak VO_2_, have similar hemodynamic responses to peak exercise. Our data suggest that fitness is a key underlying determinants of hemodynamics responses in individuals with MS.

## 1 INTRODUCTION

Multiple Sclerosis (MS) is a chronic, progressive disease of the central nervous system that begins in early adulthood and is a leading cause of morbidity and disability in young adults (Reich et al., 2018; GBD 2016 Multiple Sclerosis Collaborators, 2019) culminating in a threefold increased risk of all‐cause mortality (Brown et al., 2019; Palladino et al., 2020; Titcomb et al., 2022).

Cardiovascular disease is a significant contributor to the increased mortality in this population. Individuals with MS have nearly twice the risk of cardiovascular mortality as those without MS (Marrie et al., 2015; Palladino et al., 2020; Persson et al., 2020). Higher cardiovascular disease risk and subsequent cardiovascular function are not well understood in the population and are likely multifactorial (Mincu et al., 2018).

Individuals with MS typically also have low aerobic capacity. Aerobic capacity, measured as peak VO_2_, requires the cardiovascular, pulmonary, and musculoskeletal systems to work in concert and supply the increased demand of exercise, thus providing a whole‐body functional measure that serves as a marker of total health (Imboden et al., 2018; Ross et al., 2016). Lower peak VO_2_ in this population is partly due to disease severity, as peak VO_2_ is directly related to MS severity (Langeskov‐Christensen et al., 2015; Motl et al., 2008; Sandroff et al., 2015). However, peak VO_2_ was lower in those with self‐reported mild disease (partly defined as without walking restrictions) compared to those without MS (Klaren et al., 2016); suggesting disability does not fully explain lower peak VO_2_.

Peak VO_2_ is the product of heart rate (HR), stroke volume (SV), and arterio‐venous oxygen difference (A‐VO_2_) (Levine, 2008). During maximal exercise, individuals with MS have shown a blunted peak HR response (Klaren et al., 2016), which should lead to decreased cardiac output (Q), thus reducing peak VO_2_. Additionally, it has been suggested that individuals with MS may have compromised mitochondrial function (Kent‐Braun et al., 1997). Furthermore, cardiac function is also likely compromised in this population (Mincu et al., 2018). Therefore, one would expect all aspects that contribute to peak VO_2_ to be altered in MS. However, to our knowledge, there are no published data on SV, Q, or systemic A‐VO_2_ in response to maximal aerobic exercise in this population.

If the MS disease process is the main contributor to altered hemodynamics and A‐VO_2_ response during exercise in this population, then the alterations would be expected in people with MS with similar fitness levels as people without MS. However, if hemodynamic and A‐VO_2_ response is similar between similar fit groups of individuals with and without MS, that would suggest fitness is likely culprit of the alterations – rather than the MS disease process. Therefore, the purpose of this study was to compare the hemodynamic response to peak exercise between individuals with and without MS who have similar aerobic capacities.

## 2 METHODS

### 2.1 Participants

We recruited healthy ambulatory individuals, who were able to pedal an upright cycle ergometer, were not pregnant, nor current smokers, had no history or cardiac or pulmonary disease, and with a body mass index (BMI) less than 40 kg·m^2^ for this study. Individuals with MS received medical clearance to participate in the study and about half (11/21) of participants with MS reported exercise frequency of at least twice weekly for 30 min. The sample (*N* = 51) consisted of 21 individuals with a stable, physician‐confirmed diagnosis of relapsing–remitting MS, and 30 individuals without MS (CON). All 21 individuals with MS are included in the final analyses. Nine CON were excluded from data analyses; one due to poor echocardiography image quality and the eight youngest participants (25 ± 3 years) to more closely approximate the age of participants with MS. Thus, 42 participants were included in the final analyses, MS = 21 and CON = 21.

Disease severity was assessed with the Kurtzke's Expanded Disability Status Scale (EDSS), and inclusion criteria for this study were scores of ≤6.0 (Goldman et al., 2010; Rudick et al., 2010).

### 2.2 Study design

Volunteers arrived postprandial (≥3 h) and without caffeine or exercise for at least 24 h. After informed consent, health history questionnaire and EDSS assessment (if applicable), participants weight (kg) and height (cm) were recorded. Next, the participant rested in the supine position for at least 10 min, during which aortic diameter was measured at the aortic annulus in the parasternal long‐axis view using echocardiography (Prosound Alpha 7, Hitachi‐Aloka; Tokyo, Japan) (Lang et al., 2015; Mitchell et al., 2019). Finally, baseline heart rate (HR) was obtained with Polar v800 heart rate monitor (Polar Electro Oy, Kempele, FI). Participants were then moved to the seated position for baseline blood pressure (systolic blood pressure [SBP] and diastolic blood pressure [DBP]) and stroke volume (SV) measurements via standard sphygmomanometry and continuous wave Doppler echocardiography measurements, respectively. Mean arterial pressure (MAP) was calculated as:

$$\mathrm{MAP}=\mathrm{DBP}+\frac{1}{3}\left(\mathrm{SBP}-\mathrm{DBP}\right).$$

Peak VO_2_ was measured via an incremental exercise test on a cycle ergometer with open‐circuit spirometry system (TrueOne, Parvo Medics, Sandy, UT) to analyze expired gases. Exercise testing followed standard guidelines (American College of Sports M, 2018; Balady et al., 2010; Myers et al., 2009; Rodgers et al., 2000) with a protocol designed for persons with MS (Klaren et al., 2016; Motl & Fernhall, 2012); detailed protocol information provided in Appendix S1. BP via manual sphygmomanometer and rate of perceived exertion (RPE) were obtained during the first stage of exercise (15 Watts), and every other minute thereafter. The highest HR was recorded from the final 30 s of each minute of exercise. Stroke volume, calculated from continuous wave Doppler echocardiography measurements during seated rest (baseline), warmup, the second stage of exercise (30 watts), and then every other minute (stage) thereafter – opposite timing of BP measurements. Cardiac output (Q) was calculated as, Q=SV*HR and peak systemic arteriovenous oxygen difference (a‐VO_2_) was calculated as $\text{Peak}\;a-{\mathrm{VO}}_{2}=\frac{\text{Peak}\;{\mathrm{VO}}_{2}}{\text{Peak}\;Q}$ (De Cort et al., 1991).

### 2.3 Statistical analyses

Data are presented as mean ± standard deviation and were analyzed with Statistical Package for the Social Sciences (SPSS, version 28, IBM, Chicago, IL, USA). Alpha level was set at *p* < 0.05 to determine significance. Data were checked for normality and outliers using the Shapiro–Wilk test. In the primary analysis, MAP, SV, and Q were non‐normally distributed and corrected using the Log_10_ transformation. In the secondary analysis Age, peak VO_2_, SV, and Q were also non‐normally distributed and corrected using the log_10_ transformation. Chi‐square testing was used to examine potential sex differences between those with and without MS. Independent samples *t*‐tests were used to examine potential age, BMI, peak VO_2_, and AVO_2_ differences between groups (with and without MS [CON]). A group (MS, CON) by time (baseline, peak exercise) repeated measure analysis of variance (ANOVA) was used to evaluate changes in hemodynamic responses, included HR, MAP, SV, and Q, with maximal exercise. As the individuals with MS were significantly older than the control group, we also evaluated the hemodynamic responses via a repeated measure ANOVA in an age (and sex) matched subset (MS: *n* = 8, CON: *n* = 8) to account for age‐related differences. A one‐way ANOVA was performed to compare demographics among all four groups (MS Fit, MS Unfit, CON Fit, and CON Unfit). Then a repeated measure ANOVA was used in each group (MS and control) to compare fit and unfit individuals by time (baseline and peak exercise).

## 3 RESULTS

Descriptive characteristics are presented in Table 1. The MS group was primarily female (80%) which is consistent of the sex distribution in the population (GBD 2016 Multiple Sclerosis Collaborators, 2019; Wallin et al., 2019). Individuals with MS were older than controls, but no difference between groups in sex, BMI, peak VO_2_, or a‐VO_2_. Disease‐modifying therapy use of individuals with MS presented in Table S1.

**TABLE 1:** Descriptive characteristics of Individuals with and without MS.

	MS	Control	*p* Value
Female/Male	17/4	14/7	0.29
Age (years)	44 ± 10	31 ± 5	<0.01
BMI (kg/m^2^)	25.3 ± 4.9	25.9 ± 4.1	0.48
EDSS^b^	3.5 (2.5–4.75)	–	–
Peak VO_2_ (mL/kg*min)	28.3 ± 8.3	31.7 ± 8.2	0.58
50th percentile Peak VO_2_ (mL/kg*min^−1^)	27.8	29.9	
a‐VO_2_ (mL/100 mL)	2.4 ± 1.0	3.1 ± 1.3	0.06
Disease Modifying Therapy	16	–	
Ocrelizumab	6	–	
Natalizumab	3	–	
Ofatumumab	3	–	
Fingolimod^a^	2	–	
Diroximel fumarate	1	–	
Glatiramer acetate	1	–	
None	5	–	

*Note*: Data presented as mean ± standard deviation.

Abbreviations: a‐VO_2_, Peak systemic arteriovenous oxygen difference; BMI, Body Mass Index; EDSS, Expanded Disability Status Scale; Peak VO_2_, Peak Oxygen uptake.

^a^ Fingolimod is associated with increased risk of cardiovascular events.

^b^ Presented as median (IQR).

Baseline and peak exercise hemodynamic responses are presented in Table 2. All hemodynamic variables increased from baseline to peak exercise in both groups. There was a time*group interaction for HR, with the MS group exhibiting less HR change over time than controls (*p* < 0.01). However, there were no main group effects, although HR was nearly less in individuals with MS (*p* = 0.06). To account for potential age‐related differences in hemodynamic responses, we analyzed an age‐ and sex‐matched subset of participants. Once matched, the participants did not differ in age, BMI, peak VO_2_, or a‐VO_2_ and they exhibited similar hemodynamic responses to the entire cohort. Additionally, the time*group HR interaction was no longer significant in the subset (*p* = 0.98). Descriptive characteristics of the age and sex matched subset are presented in Table S2, and baseline and peak hemodynamic responses of the subset are presented in Table S3.

**TABLE 2:** Hemodynamic Responses at Baseline and Peak Exercise in Individuals with and without MS.

		Baseline	Peak	Time	η^2^	Group	η^2^	Interaction	η^2^
HR (bpm)	MS	64 ± 13	161 ± 15	**<0.01**	0.98	0.06	0.08	**<0.01**	0.26
	CON	59 ± 9	177 ± 11						
MAP^a^ (mmHg)	MS	86 ± 12	106 ± 13	**<0.01**	0.87	0.42	0.02	0.78	0.00
	CON	83 ± 11	103 ± 13						
SV^a^ (mL)	MS	68 ± 30	100 ± 43	**<0.01**	0.44	0.27	0.03	0.11	0.06
	CON	66 ± 29	80 ± 34						
Q^a^ (L/min)	MS	4.4 ± 2.0	13.2 ± 5.6	**<0.01**	0.88	0.37	0.02	0.86	0.00
	CON	4.0 ± 1.6	12.2 ± 6.4						

*Note*: Data presented as mean ± standard deviation. Bold vales are significant values.

Abbreviations: HR, Heart Rate; MAP, Mean Arterial Pressure; SV, Stroke Volume; Q, Cardiac Output.

^a^ Log_10_ transformed.

Secondary analysis included the same 42 participants; however, MS and CON were separated by the 50th percentile of their respective fitness levels to create MS Fit (*n* = 11), MS Unfit (*n* = 10), CON Fit (*n* = 11), and CON Unfit (*n* = 10); descriptive characteristics are presented in Table 3. The groups were different in age, BMI, and peak VO_2_.

**TABLE 3:** Descriptive Characteristics of Fit and Unfit Individuals with and without MS.

	MS		CON	
	Fit (*n* = 11)	Unfit (*n* = 10)	Fit (*n* = 11)	Unfit (*n* = 10)
Female/Male	8/3	9/1	6/5	8/2
Age^a^, ^b^ (years)	43 ± 10	45 ± 11	30 ± 4	32 ± 6
BMI (kg/m^2^)^b^	22.6 ± 3.2	28.4 ± 4.7	24.5 ± 3.0	27.4 ± 4.7
EDSS	3.0 ± 1.0	4.0 ± 1.0		
Peak VO_2_ ^a^, ^b^ (mL/kg*min)	34.3 ± 5.9	21.7 ± 4.8	38.2 ± 5.4	24.5 ± 2.7
a‐VO_2_ (mL/100 mL)	2.6 ± 1.1	2.3 ± 1.0	3.2 ± 1.4	3.1 ± 1.3

*Note*: Data presented as mean ± standard deviation. Bold vales are significant values.

Abbreviations: a‐VO_2_, Peak systemic arteriovenous oxygen difference; BMI, Body Mass Index; EDSS, Expanded Disability Status Scale; Peak VO_2_, Peak Oxygen uptake.

^a^ Log_10_ transformed.

^b^ Group differences (Fit vs. Unfit), *p* < 0.05.

Baseline and peak exercise hemodynamic responses of fit and unfit individuals both with and without MS are presented in Table 4. HR, MAP, SV, and Q increased from baseline to peak exercise in both fit and unfit groups, within their respective MS and CON groups. There was a group*time interaction for HR between fit and unfit individuals within MS, such that unfit individuals with MS had less change over time. Similarly, unfit CON had less HR change over time with a significant time*group interaction. Unfit CON almost had less change in SV from baseline to peak exercise, although the time*group interaction was not significant (*p* = 0.06). There were no main group (fit vs. unfit) effects in CON. However, in individuals with MS there was a main effect of fitness for MAP, such that fit individuals with MS had lower MAP than unfit individuals with MS and a near‐significant difference in SV; individuals with MS almost had higher SV, although the difference was not significant (*p* = 0.07).

**TABLE 4:** Hemodynamic Responses at Baseline and Peak Exercise in Fit and Unfit individuals with (MS) and without MS (CON).

			Baseline	Peak	Time	*η* ^2^	Group	*η* ^2^	Interaction	*η* ^2^
HR (bpm)	MS	Fit	59 ± 14	166 ± 11	**<0.01**	0.97	0.86	0.00	**0.04**	0.20
		Unfit	69 ± 10	157 ± 17						
	CON	Fit	54 ± 8	180 ± 9	**<0.01**	0.99	0.65	0.01	**<0.01**	0.53
		Unfit	64 ± 6	172 ± 11						
MAP (mmHg)	MS	Fit	82 ± 10	100 ± 11	**<0.01**	0.81	**0.03**	0.22	0.40	0.04
		Unfit	91 ± 14	113 ± 12						
	CON	Fit	82 ± 5	104 ± 11	**<0.01**	0.94	0.86	0.00	0.09	0.14
		Unfit	85 ± 14	103 ± 15						
SV^a^ (mL)	MS	Fit	79 ± 33	115 ± 47	**<0.01**	0.61	0.07	0.20	0.55	0.01
		Unfit	55 ± 19	84 ± 34						
	CON	Fit	78 ± 32	93 ± 35	**0.02**	0.26	0.92	0.18	0.06	0.00
		Unfit	53 ± 20	67 ± 29						
Q^a^ (L/min)	MS	Fit	4.9 ± 2.4	15.2 ± 5.8	**<0.01**	0.89	0.10	0.13	0.51	0.02
		Unfit	3.9 ± 1.4	11.0 ± 4.7						
	CON	Fit	4.5 ± 1.8	14.7 ± 7.0	**<0.01**	0.87	0.07	0.17	0.34	0.05
		Unfit	3.4 ± 1.1	9.4 ± 4.5						

^a^ Log_10_ transformed.

*Note*: Data presented as mean ± standard deviation. Bold vales are significant values.

Abbreviations: HR, Heart Rate; MAP, Mean Arterial Pressure; SV, Stroke Volume; Q, Cardiac Output.

## 4 DISCUSSION

Physical disability and reduced peak VO_2_ are typically considered interrelated consequences of MS (Klaren et al., 2016; Langeskov‐Christensen et al., 2015; Reich et al., 2018; GBD 2016 Multiple Sclerosis Collaborators, 2019). Aside from reduced peak VO_2_, little information is available regarding the physiological determinants of peak VO_2_ in those with MS. Therefore, this study investigated the hemodynamic response to peak exercise in individuals with and without MS who exhibited similar peak VO_2_, as this might provide insight on the impact of the disease process independent of fitness. Individuals with MS with similar peak VO_2_ to controls exhibited similar hemodynamic responses to peak exercise. However, when evaluating fit vs. unfit individuals with MS, our findings corresponded to the expected differences between unfit vs. fit individuals, regardless of disease status. Thus, our data indicate that the hemodynamic responses to exercise in individuals with MS are likely not due to the disease itself, but rather a result of physical inactivity or other factors affecting peak VO_2_.

At the onset of exercise, individuals with MS exhibit impaired ability to reduce vagal tone and increase HR (Hansen et al., 2013). Additionally, individuals with MS commonly have lower peak exercise HR (Klaren et al., 2016). These data suggest there is likely autonomic impairment during exercise in individuals with MS. While we did not assess HR at the onset of exercise, peak HR differences in our groups are likely explained by age. In our sub‐analysis of matched individuals with and without MS by age and sex, there was no longer a significant HR interaction. Thus, the lower HR in our group is likely due to age rather than autonomic impairment, suggesting our cohort may not exhibit autonomic impairment during dynamic exercise. Further, disease modifying therapies may impact HR and could also explain HR differences between groups (Kaplan et al., 2015). It is also possible that the HR interaction is impacted by the ability to reach a peak effort, which may differ between participants with MS and controls based on disease symptoms (i.e., coordination) (Langeskov‐Christensen et al., 2014) or fitness. Respiratory exchange ratio (RER) is a widely accepted criterion to mark peak exercise (Balady et al., 2010). RER criteria were met in most people with and without MS, and there was no difference between groups (*p* = 0.74). RER was also not significantly different between fit and unfit individuals. These RER values suggest both groups were able to achieve peak effort. However, while RER is a widely accepted as criterion for peak effort, it likely is not a demarcation of true physiologic maximal exercise (Poole et al., 2008). Although our participants reached peak exercise, they may not have achieved maximal oxygen uptake and thus a true cardiovascular maximum, which is especially challenging in special populations and unfit individuals (Poole et al., 2008). Additionally, as our exercise testing was performed with cycle ergometry, leg muscle fatigue may have been the limiting factor, not an uncommon factor in other studies (Balady et al., 2010; Kaminsky et al., 2022).

Our data show individuals with and without MS have similar MAP values. Interestingly, our secondary analyses of fitness within the MS group show fit individuals with MS have lower MAP compared to unfit individuals with MS. However, in the secondary analyses among control participants, the fit and unfit individuals were not different. This could mean fitness may have a more significant impact on blood pressure in those with MS compared to those without the disease. Considering the greater prevalence of hypertension and cardiovascular disease risk in individuals with MS, this difference highlights the importance of fitness in individuals with MS (Briggs et al., 2021; Marrie et al., 2012, 2015).

SV at rest and the response to exercise were similar between those with and without MS. When individuals with and without MS were grouped by fitness, although peak SV may appear significantly greater in the fit groups, it is simply the result of fit individuals having a higher resting SV as there was no interaction effect. This is in agreement with findings in many studies which show that well‐trained individuals have higher resting and peak SV (Rowland et al., 1997, 2002).

As expected in groups with similar peak VO_2_, peak cardiac output was not different, and there was no interaction effect. Supply must meet the demand of exercise, and since peak VO_2_ was similar, one would expect a similar peak cardiac output. Our peak cardiac output values were also similar to others (Agostoni et al., 2017; Grigoriadis et al., 2022).

Strengths of our study include the comparison of participants with and without MS with similar peak VO_2_. Examining differences in the hemodynamic response to peak exercise between individuals with and without MS, using a classic sample of low‐fit individuals compared to normal‐fit controls would make it difficult to conclude if the differential responses were due to fitness or physiologic disease process. Our MS group, while exhibiting a relatively high peak VO_2_ also included a wide range of disabilities. However, the relatively high fitness of individuals with MS who participated is unusual and different from prior studies. While our groups had similar peak VO_2_, the individuals with MS were significantly older. Also, we obtained SV using continuous wave Doppler echocardiography from the suprasternal notch, which measures maximal flow velocity along the interrogation line. Although this method is well‐accepted and has been used in many studies during exercise, flow velocity at the aortic valve may influence measurements. The same method was used in both groups in our study and is therefore directly comparable. However, caution should be taken when comparing our results with other methods of SV measurement.

## 5 CONCLUSION

Our findings show that individuals with and without MS, with similar peak VO_2_, have similar hemodynamic responses to peak exercise. The hemodynamic response to peak exercise in fit and unfit individuals with MS was comparable with the responses in fit and unfit individuals without MS. Furthermore, unfit individuals with MS have higher blood pressure compared to fit individuals with MS. However, there was not a blood pressure difference between unfit and fit individuals without MS, suggesting unfit individuals with MS may be less healthy than unfit individuals without MS. These data support the importance of fitness in this population as a key underlying correlate of hemodynamic responses to exercise. Whether randomized interventions to improve fitness in this population lead to enhanced health outcomes should be the focus of future research.

The results of the study are presented clearly, honestly, and without fabrication, falsification, or inappropriate data manipulation.

## FUNDING INFORMATION

This project was supported by the Congressionally Directed Medical Research Program through the U.S. Army Medical Research and Material Command (W81XWH1810466) and the American College of Sports Medicine Foundation (21–01556).

## CONFLICT OF INTEREST STATEMENT

Funding: Dr. Chirinos is supported by NIH grants U01‐TR003734, U01‐TR003734‐01S1, UO1‐HL160277, R33‐HL‐146390, R01‐HL153646, K24‐AG070459, R01‐AG058969, R01‐HL157108, R01‐HL155599, R01‐HL104106, and R01HL155764. He has recently consulted for Bayer, Fukuda‐Denshi, Bristol‐Myers Squibb, Johnson & Johnson, Edwards Life Sciences, Merck, and NGM Biopharmaceuticals. He received University of Pennsylvania research grants from National Institutes of Health, Fukuda‐Denshi, Bristol‐Myers Squibb, Microsoft, and Abbott. He is named as inventor in a University of Pennsylvania patent for the use of inorganic nitrates/nitrites for the treatment of heart failure and preserved ejection fraction and for the use of biomarkers in heart failure with preserved ejection fraction. He has received payments for editorial roles from the American Heart Association, the American College of Cardiology, Elsevier, and Wiley, and payments for academic roles from the University of Texas, Boston University, and Virginia Commonwealth University. He has received research device loans from Atcor Medical, Fukuda‐Denshi, Unex, Uscom, NDD Medical Technologies, Microsoft, and MicroVision Medical.

## ETHICS STATEMENT

The research protocol was approved by the University ofIllinois Chicago Institutional Review board (IRB: 2022‐0177) and allparticipants provided written informed consent. The results of the study arepresented clearly, honestly and without fabrication, falsification, orinappropriate data manipulation.

## Supporting information


Table S1.

Table S2.

Table S3.

Appendix S1.
