# Supplementary material for: Cardiovascular hemodynamic response to peak exercise in individuals with multiple sclerosis
Source: Physiol Rep. 2024 Dec 26;12(24):e70150. doi: 10.14814/phy2.70150 (PMC11671243; doi:10.14814/phy2.70150)
Supplement: Supplementary file 1 — Table S1. [file PHY2-12-e70150-s002.docx]

Supplementary Table 1. Disease Modifying Therapies of Individuals with MS

| Disease Modifying Therapy | (Trade name, Manufacturer) | n |
| --- | --- | --- |
| Ocrelizumab | (Ocrevus, Genentech) | 6 |
| Natalizumab | (Tysabri, Biogen Idec and Elan Corporation) | 3 |
| Ofatumumab | (Kesimpta, Novartis Pharmaceuticals) | 3 |
| Fingolimod^†^ | (Gilenya, Novartis Pharmaceuticals) | 2 |
| Diroximel fumarate | (Vumerity, Alkermes Pharma Ireland ltd.) | 1 |
| Glatiramer acetate | (Copaxone, Teva Pharmaceuticals) | 1 |
| None |  | 5 |

† Fingolimod is associated with increased risk of cardiovascular events.
