# Supplementary material for: Cardiovascular hemodynamic response to peak exercise in individuals with multiple sclerosis
Source: Physiol Rep. 2024 Dec 26;12(24):e70150. doi: 10.14814/phy2.70150 (PMC11671243; doi:10.14814/phy2.70150)
Supplement: Supplementary file 2 — Table S2. [file PHY2-12-e70150-s001.docx]

Supplementary Table 2. Descriptive Characteristics of Age and Sex-Matched Subset.

|  | MS | Control | *p* value |
| --- | --- | --- | --- |
| Female / Male | 6 / 2 | 6 / 2 | 0.99 |
| Age (years) | 34 ± 6 | 34 ± 6 | 0.98 |
| BMI (kg/m^2^) | 25.3 ± 5.6 | 25.9 ± 3.4 | 0.09 |
| EDSS | 3.0 ± 1.0 | -- | -- |
| Peak VO_2_^†^ (mL/kg*min^-1^) | 28.9 ± 7.4 | 27.9 ± 5.8 | 0.50 |
| a-VO_2_ (mL/100mL) | 2.6 ± 1.3 | 3.2 ± 1.4 | 0.64 |

† Reciprocal transformed

Data presented as mean ± standard deviation. BMI: Body Mass Index, EDSS: Expanded Disability Status Scale, Peak VO_2_: Peak Oxygen uptake, a-VO_2_: Peak systemic arteriovenous oxygen difference.
