# Supplementary material for: Cardiovascular hemodynamic response to peak exercise in individuals with multiple sclerosis
Source: Physiol Rep. 2024 Dec 26;12(24):e70150. doi: 10.14814/phy2.70150 (PMC11671243; doi:10.14814/phy2.70150)
Supplement: Supplementary file 3 — Table S3. [file PHY2-12-e70150-s004.docx]

Supplementary Table 3. Baseline and Peak Exercise Hemodynamic Responses in the Age and Sex Matched Subset.

|  |  | *n* | Baseline | Peak | Time | η^2^ | Group | η^2^ | Interaction | η^2^ |
| --- | --- | --- | --- | --- | --- | --- | --- | --- | --- | --- |
| HR (bpm) | MS | 8 | 64 ± 15 | 171 ± 15 | **< 0.01** | 0.98 | 0.98 | 0.00 | 0.64 | 0.02 |
|  | CON | 8 | 63 ± 6 | 173 ± 13 |  |  |  |  |  |  |
| MAP^†^ (mmHg) | MS | 8 | 87 ± 9 | 108 ± 3 | **< 0.01** | 0.88 | 0.14 | 0.15 | 0.99 | 0.00 |
|  | CON | 8 | 82 ± 15 | 99 ± 16 |  |  |  |  |  |  |
| SV* (mL) | MS | 8 | 66 ± 38 | 98 ± 54 | **< 0.01** | 0.39 | 0.35 | 0.06 | 0.37 | 0.06 |
|  | CON | 8 | 58 ± 23 | 71 ± 29 |  |  |  |  |  |  |
| Q (L/min) | MS | 8 | 4.1 ± 1.8 | 13.2 ± 6.4 | **< 0.01** | 0.75 | 0.36 | 0.06 | 0.38 | 0.06 |
|  | CON | 8 | 3.6 ± 1.3 | 10.5 ± 5.2 |  |  |  |  |  |  |

^†^Reciprocal transformed, *Log_10_ transformed

Data presented as mean ± standard deviation. HR: Heart Rate, MAP: Mean Arterial Pressure, SV: Stroke Volume, Q: Cardiac Output.
