# Supplementary material for: Cardiovascular hemodynamic response to peak exercise in individuals with multiple sclerosis
Source: Physiol Rep. 2024 Dec 26;12(24):e70150. doi: 10.14814/phy2.70150 (PMC11671243; doi:10.14814/phy2.70150)
Supplement: Supplementary file 4 — Appendix S1. [file PHY2-12-e70150-s003.docx]

**Detailed Protocol Information**

Relapsing-remitting MS is the most common subtype of the disease, comprising about 85% of patients with MS (1). Stable MS was defined as being free from neurologic episodic relapse for at least thirty days and no change in disease modifying therapy for at least six months

Aerobic Capacity protocol: Participants with and without MS performed the same protocol, beginning with a 3-minute warm-up at 0 Watts, followed by a 15 Watt/min load increase until volitional exhaustion (defined as being unable to maintain 60 revolutions per minute on the cycle ergometer, despite verbal encouragement). Peak effort was determined using standardized criteria, defined as satisfying two of the three following criteria: respiratory exchange ratio ≥1.10, peak HR within 10 beats per minute of age-predicted maximum, or peak rating of perceived exertion (RPE) ≥17 (2, 3). Peak VO_2_ was averaged using 30 second epochs, using the highest recorded value.

Continuous Wave Echocardiography: Doppler measurement of aortic blood flow velocities was performed with high-fidelity ultrasound (Prosound Alpha 7, Hitachi-Aloka; Tokyo, Japan) and 2.0 MHz continuous Doppler probe. The probe was placed at the suprasternal notch and angled until clear flow velocity waveforms were visualized, as previously described (4-6). Flow velocity images of 3-5 heart beats were traced to obtain the average velocity-time integral (VTI). Stroke volume was then calculated as the product of aortic cross-sectional area: $\pi{(\frac{aortic diameter}{2})}^{2}$ and VTI, such that $SV= \pi{(\frac{aortic diameter}{2})}^{2}*VTI.$.

**References**

1. **Dendrou CA, Fugger L, and Friese MA**. Immunopathology of multiple sclerosis. *Nature reviews Immunology* 15: 545-558, 2015.

2. **Klaren RE, Sandroff BM, Fernhall B, and Motl RW**. Comprehensive Profile of Cardiopulmonary Exercise Testing in Ambulatory Persons with Multiple Sclerosis. *Sports medicine (Auckland, NZ)* 46: 1365-1379, 2016.

3. **Motl RW, and Fernhall B**. Accurate prediction of cardiorespiratory fitness using cycle ergometry in minimally disabled persons with relapsing-remitting multiple sclerosis. *Archives of physical medicine and rehabilitation* 93: 490-495, 2012.

4. **Rowland T, Heffernan K, Jae SY, Echols G, and Fernhall B**. Tissue Doppler assessment of ventricular function during cycling in 7- to 12-yr-old boys. *Med Sci Sports Exerc* 38: 1216-1222, 2006.

5. **Rowland T, Unnithan V, Fernhall BO, Baynard T, and Lange C**. Left ventricular response to dynamic exercise in young cyclists. *Medicine and science in sports and exercise* 34: 637-642, 2002.

6. **Rowland T, and Whatley Blum JJAJoHBTOJotHBA**. Cardiac dynamics during upright cycle exercise in boys. 12: 749-757, 2000.
